# Novel grafting technique using femoral subcutaneous tissue in the surgical management of Peyronie's disease: A case report

**DOI:** 10.1016/j.ijscr.2025.111447

**Published:** 2025-05-14

**Authors:** Pande Made Wisnu Tirtayasa, I Gede Yogi Prema Ananda, Marshal Harvy Wicaksono Pantjoro, Gede Wirya Kusuma Duarsa

**Affiliations:** a Department of Urology, Faculty of Medicine, Universitas Udayana, Denpasar, Bali, Indonesia; b Prof. Dr. I.G.N.G. Ngoerah Hospital, Denpasar, Bali, Indonesia; c Universitas Udayana Teaching Hospital, Badung, Bali, Indonesia

**Keywords:** Peyronie's disease, Graft, Femoral subcutaneous tissue

## Abstract

**Introduction:**

Peyronie's disease (PD) is characterized by fibrous plaque formation in the tunica albuginea, leading to penile curvature, painful erections, and erectile dysfunction (ED). Surgical treatment often requires grafting post-plaque excision to restore function. Although various graft materials are used, the use of femoral subcutaneous tissue for PD has not been documented yet. The objective is to explore the feasibility and potential benefits of this new graft material as an alternative for PD patients.

**Case presentation:**

A 52-year-old male with Peyronie's disease presented with penile curvature and painful erections. Physical examination revealed a penile plaque. After plaque excision, a femoral subcutaneous graft was placed. Postoperatively, the patient experienced no complications, including graft rejection, erectile dysfunction, or penile shortening, and resumed normal sexual function.

**Discussion:**

Femoral subcutaneous tissue was selected for its high vascularity, ease of harvesting, and low complication risk. Compared to commonly used grafts, it offers better perfusion and tissue integration, reducing the risk of PD recurrence and shortening.

**Conclusion:**

This case highlights the successful utilization of femoral subcutaneous tissue as a graft for PD surgery, offering promising results. Further research is needed to validate its long-term efficacy compared to traditional grafts.

## Highlights

• Tirtayasa-Duarsa technique is presented as a novel autologous graft option for Peyronie’s disease surgery.
• The procedure achieved satisfactory penile curvature correction while preserving erectile function.
• This technique offers potential benefits in biocompatibility and reduced complications.

## 1 Introduction

Peyronie's disease (PD) is a connective tissue disorder affecting the tunica albuginea of the penis. It features a formation of inelastic fibrous plaques caused by abnormal wound healing. These plaques result in penile curvature, painful erections, erectile dysfunction (ED), and difficulties with penetration [1,2]. The self-reported prevalence of PD was 0.5–13 %, with 33 % of them reported discomfort while engaging in sexual intercourse [3]. PD can affect the quality of sexual experience and the quality of life in general. PD was also linked with anxiety, low self-esteem, and depression [4,5].

According to the American Urological Association (AUA) guideline, management of PD includes oral medications, intralesional injections, or surgical interventions depending on the stage of the disease [6]. Medical therapies are primarily utilized in the early phases of PD, while surgical treatment is reserved for the chronic stage when deformities become severe or unresponsive to medical treatment [7]. Surgery is considered the gold standard for correcting PD where penile curvature persists for more than a year, plaques remain stable for over 3 months, sexual function is impaired due to curvature, or significant penile shortening and aims to restore a functional penis with less than 20 degrees of curvature [8,9].

Surgical intervention often involves the use of grafts to cover the exposed tissue following plaque excision. A variety of graft materials have been used, such as autologous grafts, allografts, xenografts, and synthetics [6,10]. Autologous grafts have traditionally included tissues such as dermis, tunica vaginalis, tunica albuginea, dura mater, fascia, saphenous vein, and buccal mucosa [11]. To our best knowledge, the use of femoral subcutaneous tissue as a graft material has not yet been documented in the context of PD surgery. Our aim is to explore the feasibility and potential benefits of this method, which may offer an alternative solution for patients undergoing surgical correction of PD.

## 2 Case presentation

A 52 years old male came to the outpatient clinic with a chief complaint of penile curvature. He also had a painful erection for 2 months. He experienced difficulty with sexual intercourse due to dorsal penile curvature during erection. The patient denied any history of trauma to the penis. During physical examination, a palpable plaque sized approximately 1 cm × 2 cm in the dorsal penis was identified. Doppler ultrasound of the penis confirmed the presence of a fibrous plaque on the dorsal surface of the tunica albuginea, consistent with Peyronie's disease. Given the sexual impairment caused by the penile deformity, surgical correction was indicated in this patient.

**Fig. 1:**
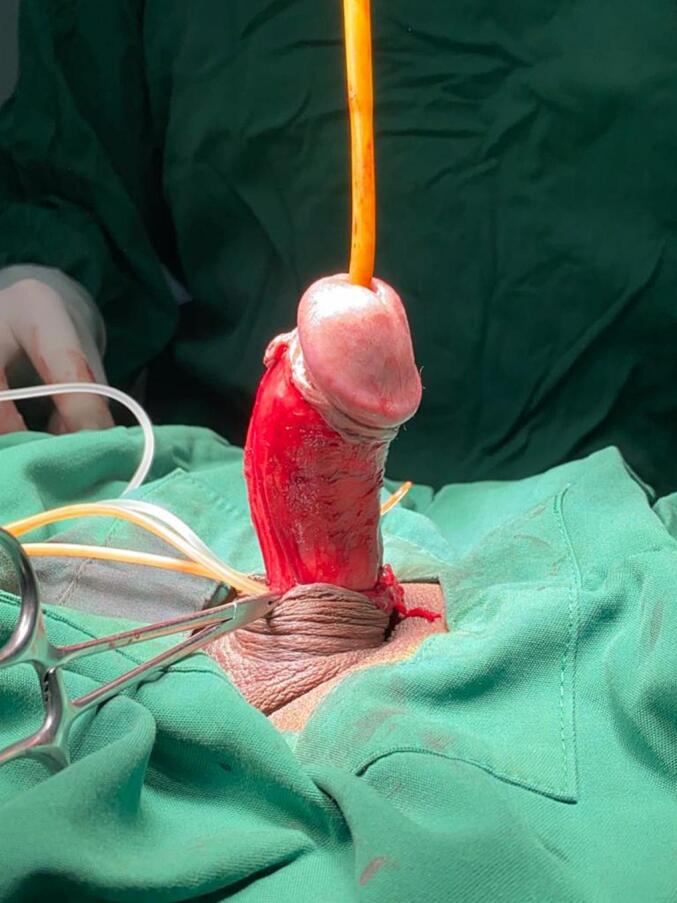
Dorsal curvature after intracavernosal injection with normal saline.

**Fig. 2:**
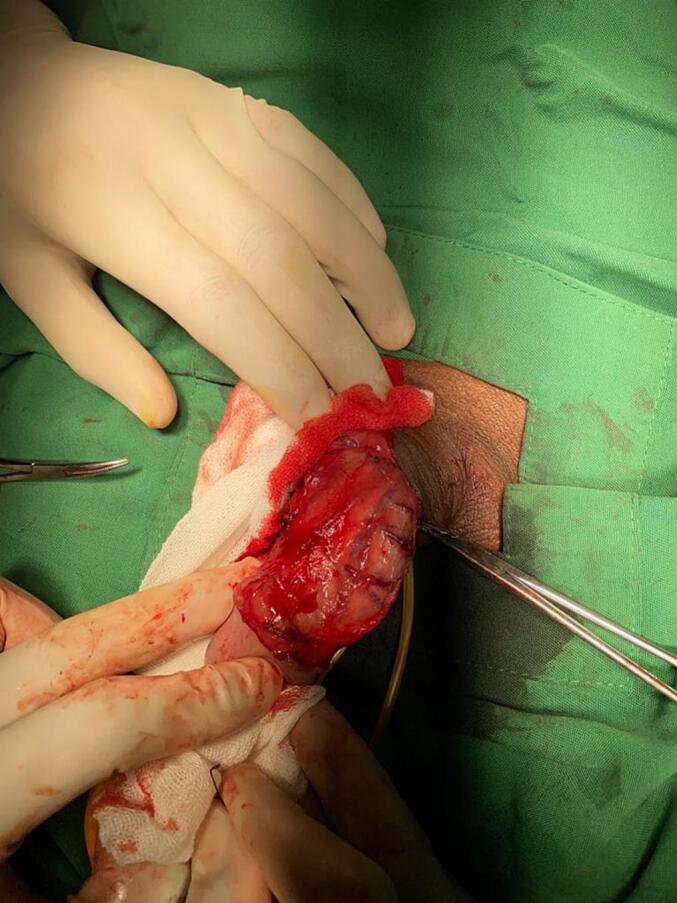
Plaque exposure.

**Fig. 3:**
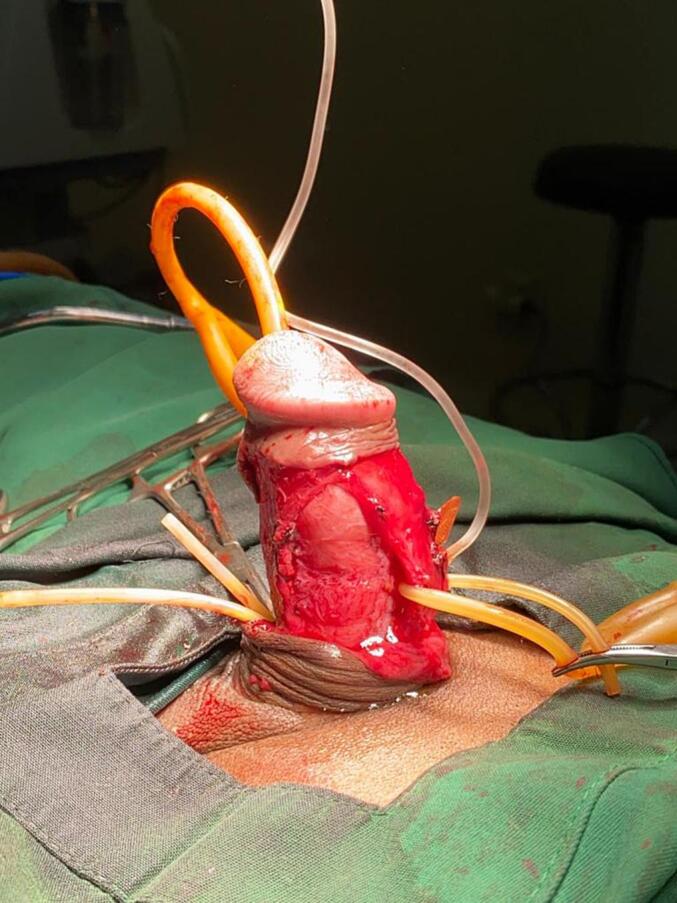
Post dorsal plaque excision.

The patient was prepared for plaque excision and grafting using the Tirtayasa-Duarsa technique, incorporating femoral subcutaneous tissue as a novel grafting material—a method not previously reported in the literature for the treatment of Peyronie's disease. The surgery started with circumcising and degloving the entire penile shaft by opening Buck's fascia on both sides. Because of the dorsal location of the plaque, the neurovascular bundle was dissected and separated carefully to avoid injury. An artificial erection by intracavernosal injection of normal saline was induced to identify the point of maximum curvature, which would mark the point for the surgeon to do a plaque excision (Fig. 1). A small incision was made at the point of maximum curvature to allow the concave side of the penis to stretch out and reduce the curvature. The cut was carefully extended laterally to relieve the tension caused by the plaque (Fig. 2). It was done carefully to avoid damaging the underlying erectile tissues (Fig. 3).

**Fig. 4:**
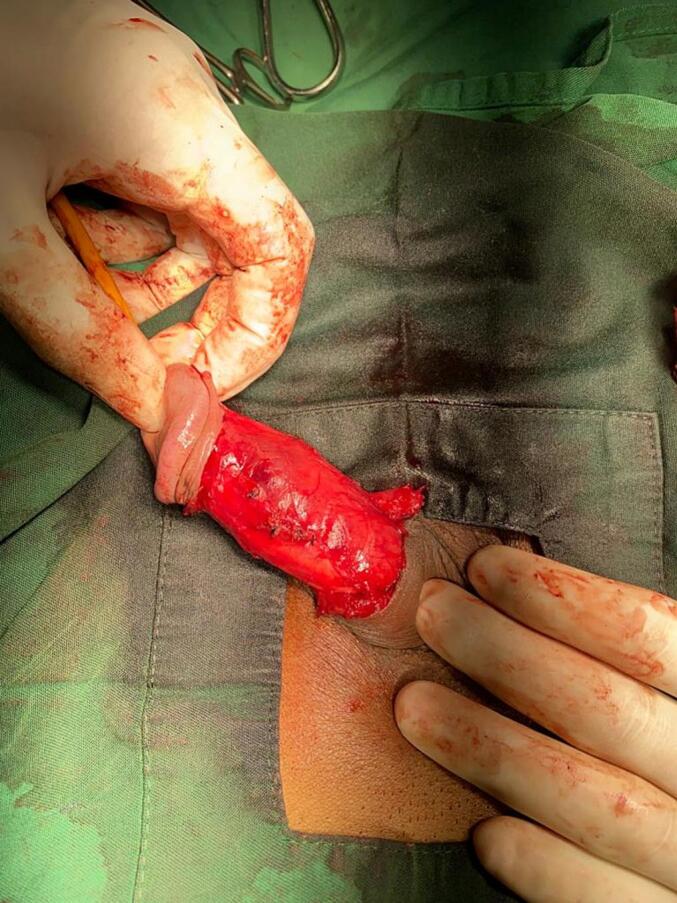
Post grafting using femoral subcutaneous tissue.

**Fig. 5:**
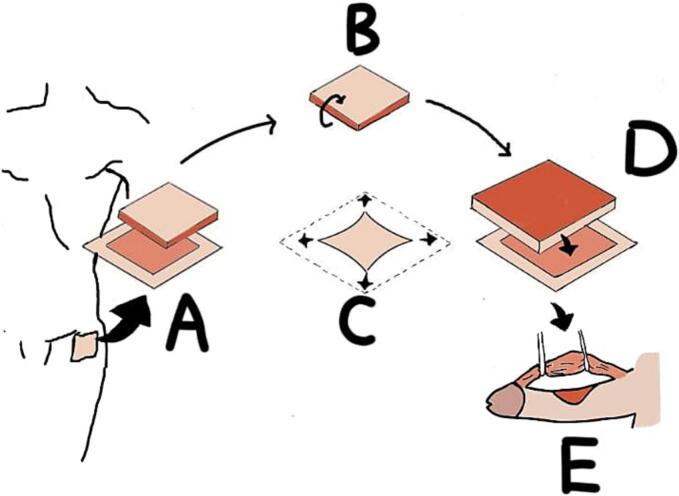
Illustration of the Tirtayasa-Duarsa technique (A. harvesting the subcutaneous femoral tissue from the left upper thigh below the anterior superior iliac spine; B. flipping the subcutaneous femoral tissue to expose the side with high vascularity; C. stretching the subcutaneous femoral tissue to fully cover the defect; D. grafting the subcutaneous femoral tissue into the excised penile defect; E. retracting the neurovascular bundle to avoid injury to those structures).

Following excision, the exposed penile tissue was prepared for the placement of an autologous graft harvested from the patient's femoral subcutaneous tissue. The donor site was marked with an additional 0.5 cm margin beyond the plaque size to ensure adequate graft coverage. The excision extended to the highly vascular femoral subcutaneous layer, and the graft was inverted to position the vascular surface facing upward to ensure vascularization of the penile skin and protect the exposed tunica albuginea after plaque removal. The graft was sutured in place with continuous suture, ensuring smooth coverage and proper integration (Fig. 4). The complete step of the Tirtayasa-Duarsa technique was illustrated in Fig. 5. After the surgery and graft placement were completed, intracavernosal injection testing with normal saline was repeated, showing improvement in penile curvature during erection. Then the Buck's fascia was closed on both sides. After that, penile skin was closed with interrupted sutures. The total duration of this surgery was 97 min. Eventually, the patient was discharged and observed through the outpatient clinic.

Postoperatively, the patient was followed up at 3 months, 6 months, and 1 year. Throughout this period, there were no signs of graft rejection, infection, or plaque recurrence. The patient expressed high satisfaction and reported significant improvement in quality of life. Additionally, no complications such as erectile dysfunction (ED), glans hypoesthesia, or penile shortening were noted. He was able to resume normal sexual activity with minimal residual curvature and no erection pain. The use of femoral subcutaneous tissue as a graft proved to be successful, with excellent graft integration and no postoperative complications. This case has been reported in accordance with the SCARE criteria to ensure a standardized and comprehensive presentation [12].

## 3 Discussion

Peyronie's disease (PD) is a connective tissue disorder affecting the tunica albuginea, where fibroelastic plaques form due to impaired wound healing. According to the American Urological Association (AUA) guidelines, treatment options for PD range from oral medications and intralesional injections to surgical interventions, depending on the stage of the disease [6]. Surgical intervention in Peyronie's disease (PD) is indicated when penile curvature persists for over a year, plaques remain stable for more than three months, or significant penile shortening or impaired sexual function is present [9]. The potential adverse outcomes of surgical intervention includes ED, glans hypoesthesia, and penile shortening [13].

The plaque excision will always leave exposed tunica albuginea, and it needs to be covered by graft. Grafting helps avoid further penile shortening or penis with hourglass deformities. It also brought satisfaction, no significant length change, straightening, and no significant complication [11,13]. Several studies have cited different types of autologous grafts, including tunica vaginalis, tunica albuginea, saphenous vein, dermis, buccal mucosa, lingual mucosa, rectus fascia, and fascia lata [11,13,14]. To the best of our knowledge, the use of femoral subcutaneous tissue as an autologous graft in the Tirtayasa-Duarsa technique for Peyronie's disease has not been previously documented in the literature.

Femoral subcutaneous tissue was chosen based on its high vascularity, ease of access, good tissue compatibility, and low risks of infection and graft rejection. These characteristics align well with the ideal graft requirements such as availability, resistance to infection, minimal contraction, promotion of hemostasis, preservation of erectile function, cost-effectiveness, and not prolonging operative time [13]. This choice is further supported by current literature highlighting the advantages of autologous grafts, such as safety, ease of harvesting without complex techniques [15], compatibility with host tissue, reduced risk of local inflammation and infection, and cost-effectiveness [10,[16], [17], [18]]. Additionally, they have been shown to improve patients' penile appearance and sexual outcomes [19].

Two of the most commonly used graft material for Peyronie's disease are saphenous vein and lingual mucosa. Saphenous vein has been used since 1998 due to its ease of procurement, flexibility, resistance to blood pressure changes, and high compatibility with host tissue and buccal mucosa frequently used graft due to its ease of harvesting and low complication risk. However, these grafts also carry a higher risk of hypoperfusion from the endothelium, which can increase the chances of PD recurrence and penile shortening [20]. This is where the femoral subcutaneous graft has the upperhand due to its high vascularity, promoting better tissue perfusion and potentially reducing these risks. Additionally, femoral subcutaneous tissue also possesses these beneficial properties, particularly with regard to vascularization, but also offers the added benefit of high tissue compatibility and minimal risk of inflammation.

In our case, the patient reported no complications during the follow-up period, further supporting the safety of the femoral subcutaneous tissue graft. These findings underscore the potential of femoral subcutaneous tissue as a viable alternative to more commonly used grafts, particularly in cases where high vascularity and ease of harvesting are priorities. The absence of significant complications, including erectile dysfunction, glans hypoesthesia, or penile shortening, supports the safety and effectiveness of this grafting technique.

The patient also expressed high satisfaction with the surgical outcome, including the ability to resume normal sexual activity without erection pain and with only minimal residual curvature. Satisfaction was assessed through direct postoperative interviews, during which the patient reported improved confidence, sexual performance, and quality of life. However, we recognize that this assessment remains subjective, and future studies should incorporate validated tools, such as the International Index of Erectile Function (IIEF), to better quantify patient-reported outcomes.

The primary limitation of this study is that it is based on a single case report, which restricts the generalizability of the findings. Additionally, the follow-up duration of one year, while sufficient for early and intermediate outcome assessment, may be insufficient to fully evaluate long-term graft durability and late-onset complications. Extended follow-up and longitudinal studies are needed to address these aspects more thoroughly. Future research, including larger case series and controlled clinical trials, is essential to evaluate the efficacy and safety of femoral subcutaneous grafting in a broader population. Comparative studies with other established graft materials, such as saphenous vein and buccal mucosa, would further help determine its relative advantages and long-term performance.

## 4 Conclusion

The use of femoral subcutaneous tissue as a graft material in Peyronie's disease surgery presents a novel and promising alternative graft material. Its high vascularity, ease of harvesting, and minimal risk of complications make it a viable option for patients undergoing plaque excision. While early results are encouraging, further research is needed to validate its long-term efficacy and establish its role as graft material for Peyronie's disease.

## Consent

Written informed consent was obtained from the patient for the publication of his photographs in this report.

## Ethical statement

This study was deemed exempt from ethical approval by the Ethics Committee of our institution. Written informed consent was obtained from the patient for publication and any accompanying images. A copy of the written consent is available for review by the Editor-in-Chief of this journal on request.

## Guarantor

I Gede Yogi Prema Ananda.

## Funding

No financial support or grants were received by the authors for this article.

## Author contribution

**Pande Made Wisnu Tirtayasa**: Surgeon operator, study concept and design, surgical procedure, data collection, and manuscript revision.

**I Gede Yogi Prema Ananda**: Data analysis, interpretation of results, manuscript writing, and corresponding author.

**Marshal Harvy Wicaksono Pantjoro**: Literature review, manuscript writing, figure preparation and drawing.

**Gede Wirya Kusuma Duarsa**: Surgeon operator, critical revision for intellectual content and final manuscript approval.

## Declaration of competing interest

The authors declare no conflicts of interest.
